# 

**Published:** 1917-01-06

**Authors:** 


### Matrons' and Sisters' Appointments.

Elstree Red Cross Hospital, Bromley.—Miss Edith N. Constable has been appointed sister. She was trained at the Royal Hants County Hospital, Winchester, and has since been staff nurse at the National Hospital for Paralysed and Epileptic, Bloomsbury, night sister at Birmingham Ear and Throat Hospital, sister at the Devonshire Hospital, Buxton, and at the North Eastern Fever Hospital, Tottenham. Miss Constable has also been a Queen's nurse at Manchester, and holds the certificate of the Incorporated Society of Trained Masseuses.

**NOTE.—Particulars of Appointments for insertion in this column will be welcomed.**

### Hospital and Institutional Results in 1915.

**Dundee Royal Infirmary.**—The accounts of the infirmary are made up from June to June. The present is a time when the figures of one year, for many reasons, are less comparable with those of another than in normal times. We therefore put it to the Board of Directors that now occurs a favourable opportunity to consider a proposition to bring the time over which the accounts extend into line with that adopted by the vast majority of hospitals in Great Britain and Ireland. Eighteen thousand one hundred and ninety-five in-patients were treated; as compared with 19,869 in the previous twelve months. The average cost per occupied bed has gradually risen from £58 in 1912 to £70 in 1916.

**Dorset County Asylum for the Treatment of Mental Diseases**—The number of patients resident on March 31, 1916, was 1,117, being thirty more than at the end of the previous statistical year. The maintenance rate was 9s. 11d. per week, an increase of 1s. 2d. as against 1914-1915. Fat stock for slaughtering at home to the value of £4,620 was bought, and produced 120,333 lb. of meat in carcase. After the cost of food has been added and credit taken for the sale of hides, etc., the meat works out at about 8¼d. per lb.

**Western Auxiliary Military Hospital, Torquay.**—This hospital, formerly devoted to cases of incipient consumption, is now used for the wounded. The accounts are made up from October 1, 1915, to September 30, 1916, and the daily average number of patients was 75.45. As the expenditure amounted to £6,206, the average cost for the year of each bed would be a little over £82, but the accounts as presented do not admit of an accurate calculation in this respect.

**The David Lewis Manchester Epileptic Colony.**—The financial year of the colony ends on August 31. It is probable that the reason why it thus differs from the generality of institutions is quite unknown to the present management. The income for the year was £15,880, of which sum £14,192 was paid for the maintenance of the colonists; the expenditure for the same period was £14,818. The profit on general and poultry farming came to £480.

### GUY'S HOSPITAL.

In connection with the scheme lately sanctioned by the Local Government Board and the London County Council for the diagnosis and treatment of venereal disease, the following appointments for 1917 have been made at Guy's Hospital: Assistant to the obstetric surgeons, Miss Morna Rawlins, M.B., B.S. Lond.; assistant to the dermatologist, Mr. L. S. Gathergood, M.R.C.S., L.R.C.P.; assistant to the genito-urinary surgeon, Captain G. E. Genge-Andrews, M.B., B.S. Lond.; assistant bacteriologist, Miss Una Griffin, M.B., B.S. Lond.

### IMPORTANT NOTICE.

Frequently of late the Manager regrets that he has been unable to supply extra copies of The Hospital containing articles and other matter of special interest to some of its readers. The restrictions in regard to paper and the difficulties of production at the present time are responsible for the inability to meet these additional demands after publication, by rendering it necessary that the number printed of each issue be limited to actual requirements. If, however, extra copies or reprints are ordered not later than the first post on the Monday following the date of the particular issue required, arrangements can be made to satisfy the needs of all readers of The Hospital. Applications should be addressed to the Manager, The Hospital, 28 Southampton Street, Strand, London, W.C.

The Chairman of the Grimsby Hospital intimates that Mr. Alec L. Black has offered £1,000 to the institution for the endowment of a bed, which the donor suggests shall be named the "Lloyd George Bed."

Rates of Subscription: Twelve months, 6/6; Six months, 4/-; Three months, 2/-; post free to any address in the United Kingdom. Abroad, Twelve months, 8/8; Six months, 5/-; Three months, 2/6, post free. The Hospital "Index" to Volume LX., April to September 1916, is now ready, price 2½d. per copy, post free. Address The Manager, "The Hospital," The Hospital Building, 28/29 Southampton Street, Strand, London, W.C.

